# Fructose promotes ampicillin killing of antibiotic-resistant *Streptococcus agalactiae*

**DOI:** 10.1080/21505594.2023.2180938

**Published:** 2023-02-26

**Authors:** Xuan-Wei Chen, Jia-Han Wu, Ying-Li Liu, Hetron Mweemba Munang’andu, Bo Peng

**Affiliations:** a State Key Laboratory of Biocontrol, Guangdong Key Laboratory of Pharmaceutical Functional Genes, School of Life Sciences, Southern Marine Science and Engineering Guangdong Laboratory (Zhuhai), Sun Yat-sen University, Higher Education Mega Center, Guangzhou, China; b Laboratory for Marine Biology and Biotechnology, Qingdao National Laboratory for Marine Science and Technology, Qingdao, China; c Faculty of Biosciences and Aquaculture, Nord University, Bodo, Norway

**Keywords:** *Streptococcus agalactiae*, ampicillin, fructose, glycolysis, metabolism

## Abstract

*Streptococcus agalactiae* (GBS) is an important pathogenic bacteria that infected both aquatic animals and human beings, causing huge economic loss. The increasing cases of antibiotic-resistant GBS impose challenges to treat such infection by antibiotics. Thus, it is highly demanded for the approach to tackle antibiotic resistance in GBS. In this study, we adopt a metabolomic approach to identify the metabolic signature of ampicillin-resistant GBS (AR-GBS) that ampicillin is the routine choice to treat infection by GBS. We find glycolysis is significantly repressed in AR-GBS, and fructose is the crucial biomarker. Exogenous fructose not only reverses ampicillin resistance in AR-GBS but also in clinic isolates including methicillin-resistant *Staphylococcus aureus* (MRSA) and NDM-1 expressing *Escherichia coli*. The synergistic effect is confirmed in a zebrafish infection model. Furthermore, we demonstrate that the potentiation by fructose is dependent on glycolysis that enhances ampicillin uptake and the expression of penicillin-binding proteins, the ampicillin target. Our study demonstrates a novel approach to combat antibiotic resistance in GBS.

## Introduction

*Streptococcus agalactiae*, also called Group B streptococcus (GBS), is a type of Gram-positive bacteria that represents a severe pathogen to both animals and human beings [1–3]. GBS represents one of the most severe pathogens in threatening high-risk populations including neonates and elderly people [3] but also causes massive death in aquatic animals including Nile tilapia, sea bream, silver pomfret, mullet, golden shiners and bull minnows as documented in different countries [4–6], resulting in huge economic loss in aquaculture.

β-lactams is the primary choice of antibiotics to treat invasive GBS disease [7]. However, resistance to β-lactams is reported in GBS clinic isolates [8–15]. More importantly, multidrug-resistant GBS are resistant to a variety of classes of antibiotics including aminoglycosides, sulphonamides, macrolides and quinolones [15–17]. Since the lack of effective measures to control such infection by antibiotic-resistant bacteria, alternative strategies should be developed as an alternative to the current available antibiotics.

Based on the assumption that metabolic state determines antibiotic killing efficacy, recently developed reprogramming metabolomics provides a potential strategy to combat antibiotic resistance [18–20]. This strategy starts with discovery metabolomics that compare the metabolomes of antibiotic resistant bacteria with antibiotic-sensitive bacteria, from which to identify the metabolites whose abundance is repressed in the resistant bacteria. Exogenous administration of the key repressed metabolites is able to re-sensitize antibiotic-resistant bacteria to antibiotics. Glucose, alanine, fructose and glutamate reverses kanamycin- and gentamycin-resistant bacteria [21–24]; Glutamine sensitizes ampicillin to kill multidrug-resistant *Escherichia coli* through purine metabolism that generates inosine, a key regulator of OmpF expression [19]; fumarate, cysteine or thymine enhances killing efficacy of quinolones [25–27]. This strategy is not only applicable to antibiotic-resistant bacteria but also serum-resistant bacteria, where glycine reverses serum resistance to enhance complement-dependent killing [28]. However, these studies mainly focuses on Gram-negative bacteria, and Gram-positive bacteria is less explored. Here, we investigate the metabolic signature of GBS with Amp resistance and explore potential metabolite to reverse ampicillin resistance in GBS. The result suggests that fructose is a potential metabolite on reverting antibiotic resistance depending on glycolysis. Data are shown below.

## Results

### GBS mounts metabolic shift towards Amp resistance

**Figure 1.**
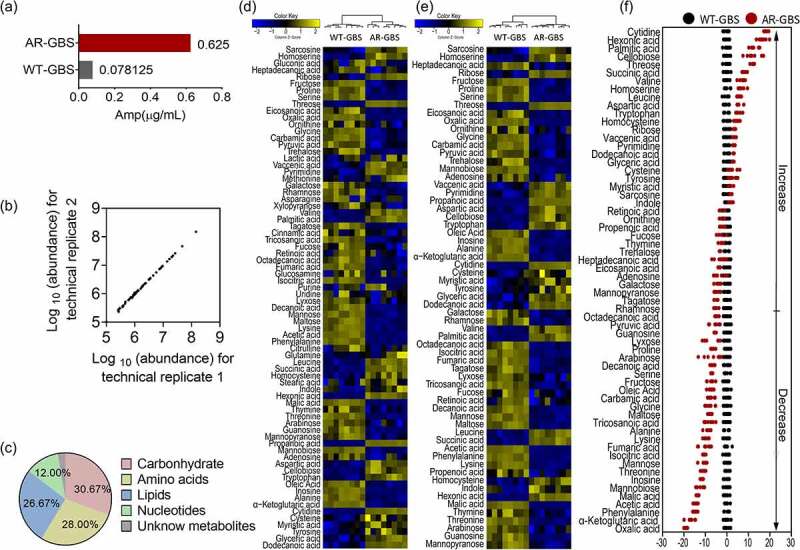
Metabolomic analysis of WT-GBS and AR-GBS (a) MIC between WT-GBS and AR-GBS. (b) Reproducibility of metabolomic data. (c) Categories of identified differential metabolites. (d) Heat map showing relative abundances of metabolites in AR-GBS and WT-GBS. Yellow and blue indicate increased and decreased metabolite levels relative to the median metabolite level, respectively (see the colour scale). (e) Heatmap showing relative abundances of differential metabolites between AR-GBS and WT-GBS. Yellow and blue indicate increased and decreased metabolite levels relative to the median metabolite level, respectively (see the colour scale). (f) Z-score plots (e). The data from AR-GBS are separately scaled to the mean and standard deviation of WT-GBS. Each point represents replicate and colour indicating sample type. Black and Red represent WT-GBS and AR-GBS, respectively.

To dissect the metabolic characteristics underlying GBS ampicillin (Amp) resistance and searching for potential metabolites to reverse the antibiotic resistance, we generated the GBS-Amp resistant strains (AR-GBS) by sequentially propagating the wild type strain (WT-GBS) in gradual increased MIC as previously described [22]. The resulted AR-GBS resistant strain was confirmed by MLST [29]. At last, the AR-GBS resistant strain had 8 folds of increased MIC value than WT-GBS (Figure 1(a)). WT-GBS and AR-GBS, cultured in medium without antibiotics, were collected for Gas chromatography-mass spectrometry (GC-MS)-based metabolomic analysis. Four biological replicates were included for each strain, and two technique replicates were included for each biological replicate. Thus, 16 data points were obtained. The Pearson correlation coefficients were 99.98% ± 99.99% with the relative standard deviation was 0.70%, demonstrating the reproducibility of the data (Figure 1(b)). In total, 234 peaks were identified, and the metabolites were characterized via NIST database. Finally, after removing the internal standard and known solvent peaks, 75 non-redundant metabolites were obtained. Based on the functional categories, the 75 metabolites fell in the categories of carbohydrate (30.67%), amino acids (28.00%), lipids (26.67%), nucleotides (12.00%) and unknown metabolites (2.67%), respectively (Figure 1(c)). The metabolites were displayed as a heatmap in Figure 1(d). Metabolites of differential abundance were obtained by Kruskal-Wallis test (Wilcoxon rank sum test), where 62 metabolites were of differential abundance. As compared to WT-GBS, the false discovery rate was 0.70% (Figure 1(e)). z-score spanned from −6.63 to 89.50 in the AR-GBS group, wherein the abundance of 22 metabolites were increased and 40 metabolites were decreased (Figure 1(f)). All together, these results suggest that AR-GBS adjusted its metabolism upon Amp resistance.

### Pathway enrichment analysis

**Figure 2.**
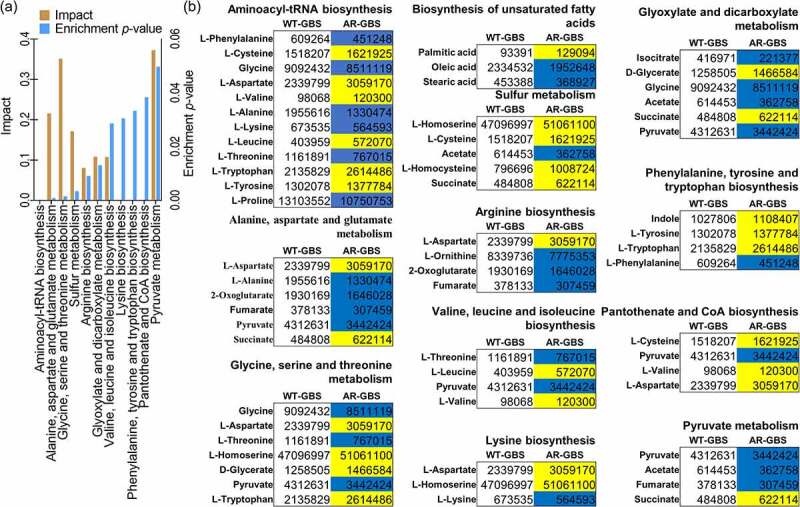
Biomarkers and pathways differentiating WT-GBS and AR-GBS. (a) Pathway enrichment analysis. (b) the volume of the differential metabolites of AR-GBS compared with WT-GBS. The colour of blue indicates metabolites of decreased abundance; yellow indicates metabolites of increased abundance.

To investigate the metabolic networks that were involved in Amp resistance, 66 differential metabolites were searched against an on-line tool, MetaboAnalyst 5.0 [30]. Twelve pathways were identified, including aminoacyl-tRNA biosynthesis, alanine, aspartate and glutamate metabolism, glycine, serine and threonine metabolism, sulphur metabolism, arginine biosynthesis, glyoxylate and dicarboxylate metabolism, valine, leucine and isoleucine biosynthesis, lysine biosynthesis, phenylalanine, tyrosine and tryptophan biosynthesis, pantothenate and CoA biosynthesis and pyruvate metabolism (Figure 2(a)). Interestingly, the abundance of four out of six metabolites, four out of five metabolites, three out of four metabolites and three out of four metabolites was decreased in alanine, aspartate and glutamate metabolism, citrate cycle, arginine biosynthesis and pyruvate metabolism, respectively (Figure 2(b)). This enrichment analysis indicated that these metabolic pathways were severely impaired in AR-GBS bacteria. More importantly, these pathways were interconnected with each other via citrate cycle in most of the bacteria [31,32]. However, Streptococcus does not contain a fully functional citrate cycle (Supplementary Figure S1) [33]. In considering the decreased abundance of pyruvate and 2-oxoglutarate that drives the formation of alanine and glutamate, and reduced abundance of fumarate that connects with arginine biosynthesis. We speculate that the upstream metabolic pathway, glycolysis, are impaired.

### Glycolytic pathway is repressed in AR-GBS

**Figure 3.**
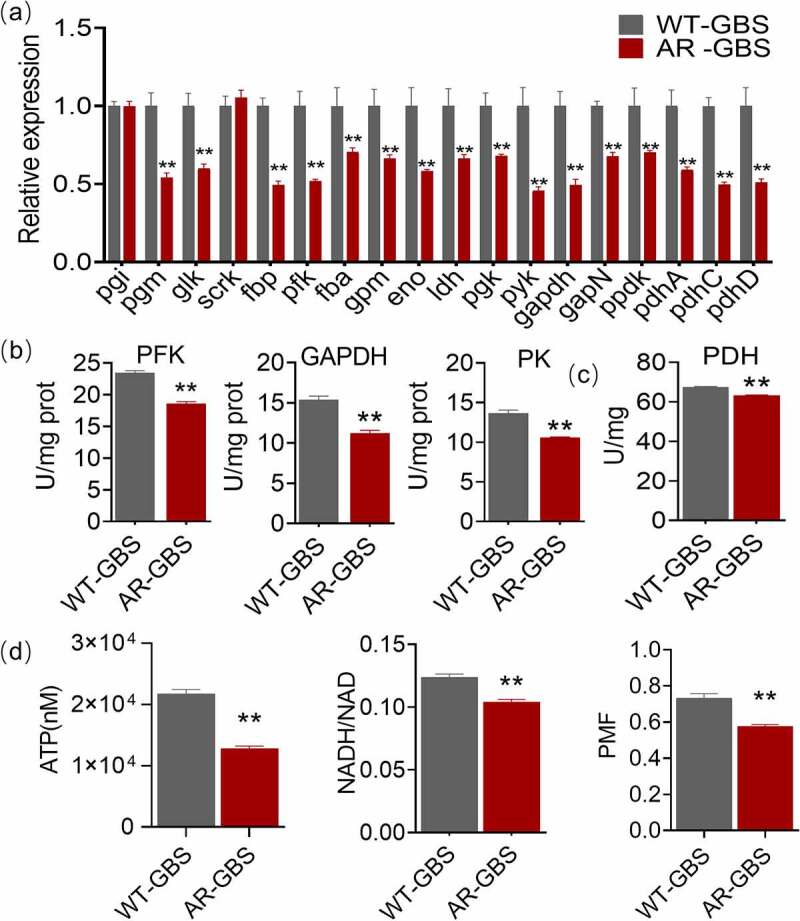
Glycolysis is impaired in AR-GBS (a) Real-time quantitative reverse transcription-PCR (qRT-PCR) for genes expression of the glycolysis. (b, c) Activity of PFK, GAPDH, PK and PDH of WT and AR in 5 ml 25% BHI medium (OD600 = 0.2) at 37 °C for 6 h. (d) ATP, NADH and Membrane potential (PMF) of WT and AR in 5 ml 25% BHI medium (OD600 = 0.2) at 37 °C for 6 h. Results are displayed as mean ± standard errors of the means (SEM) (*N* ≥ 3 technical replicates per sample), and statistically significant differences are identified by t-test. *, *p* < 0.05, **, *p* < 0.01. Each experiment was repeated independently at least three times.

To explore whether glycolysis was involved in Amp resistance, the expression of glycolytic genes including *pgi*, *pgm*, *glk*, *scrk*, *fbp*, *pfk*, *fba*, *gpm*, *eno*, *ldh*, *pgk*, *pyk*, *gapdh*, *gapN*, *ppdk*, *pdhA, pdhC* and *pdhD* were quantified in WT-GBS and AR-GBS (Figure 3(a)). Interestingly, the expression of all of the genes was downregulated except that of pgi and scrk. Specifically, *fbp*, *pfk*, *gapdh*, *pyk*, *pdhC* and *pdhD* were decreased around two folds in AR-GBS (Figure 3(a)). To confirm this data at protein level, specific enzyme activity of phosphofructokinase (PFK), glyceraldehyde-3-phosphate dehydrogenase (GAPDH) and pyruvate kinase (PK) encoded by *pfk*, *gapdh* and *pyk*, respectively, were quantified. Being consistent with the gene expression data, activities of PFK, GAPDH and PK were reduced 1.26, 1.37 and 1.29 folds in AR-GBS, respectively (Figure 3(b)). Decreased glycolysis would lower the pyruvate and the downstream metabolic pathways. The specific enzyme activity of pyruvate dehydrogenase (PDH), catalysing the formation of acetyl-CoA from pyruvate, was decreased accordingly (Figure 3(c)). Glycolysis plays critical roles in generating ATP and NADH, whose content were decreased in AR-GBS (Figure 3(d)). NADH is as the donor for the generation of proton motif force (PMF), whose level was also decreased in AR-GBS. These data together suggest that glycolysis was decreased in AR-GBS.

### Multivariate analysis

**Figure 4.**
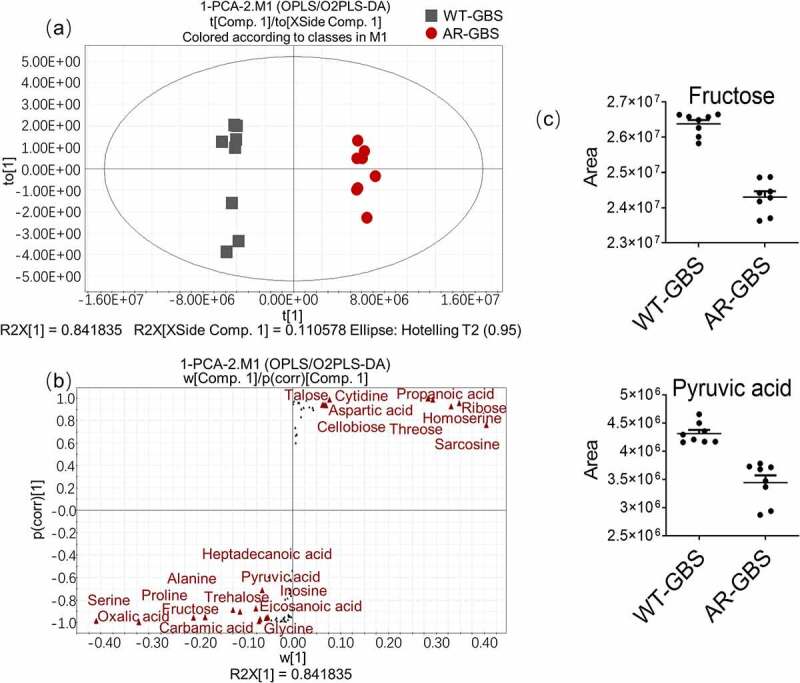
Multivariate analysis of metabolomic data (a) PCA score plot. Each dot corresponds to an individual sample. Black and Red represent WT-GBS and AR-GBS respectively. (b) S-plot. Each circle represents individual metabolite, which is greater or equal to 0.05 and 0.5 for the absolute value of covariance p and correlation p(corr) respectively. (c) Scatter plot showing the relative abundance of fructose and pyruvate from data (b).

In searching for the metabolite(s) that can reverse the Amp resistance in AR-GBS, multivariate analysis was carried out to identify the crucial biomarkers that contribute to the differential metabolism between WT-GBS and AR-GBS. Thus, orthogonal partial least-squares discriminate (OPLS-DA), the supervised multiple regression analysis, was adopted. WT-GBS and AR-GBS was clearly separated from each other (Figure 4(a)), indicating these two groups had different metabolic signature. Then, S-plot demonstrated the discriminating variables of the two groups shown in Figure 4(b), where the red triangles highlighted the differential metabolites having the largest weights (< − 0.05 or>0.05) and highest relevance (< − 0.05 or>0.05). At last, a total of 18 crucial biomarkers were identified as shown in Figure 4(c) & Supplementary Figure S2. To select the potential biomarkers to reverse Amp resistance, we integrated the pathway enrichment analysis and the identified crucial biomarkers. As suggested from pathway enrichment data, we speculate glycolysis is involved. And the abundance of crucial biomarker, pyruvate and fructose belonging to glycolysis, were all decreased in AR-GBS. Since fructose fuels glycolysis, it was selected as a potential biomarker to reverse Amp resistance.

### Fructose potentiates Amp to kill AR-GBS

**Figure 5.**
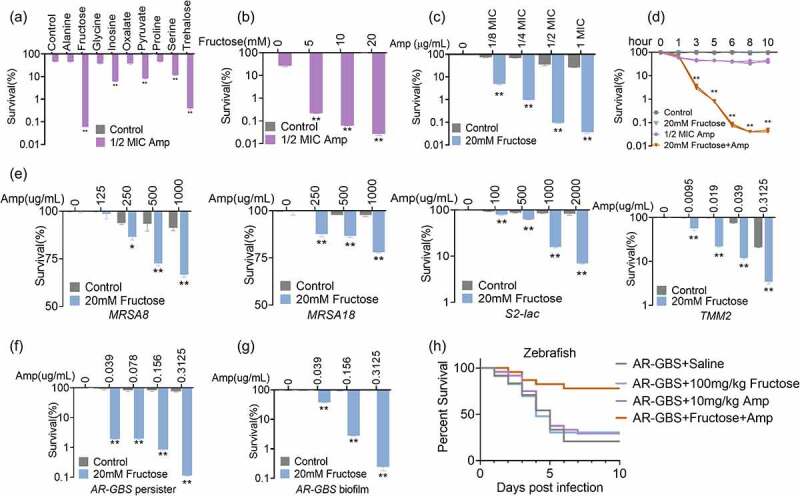
Fructose potentiates ampicillin to kill AR-GBS. (a) Percent survival of AR-GBS in the presence of the indicated metabolites plus Amp. (b) Percent survival of AR-GBS in the indicated concentrations of fructose plus 1/2 MIC Amp. (c) Percent survival of AR-GBS in the indicated concentrations of Amp plus 20 mM fructose. (d) Percent survival of AR-GBS in 20 mm fructose plus 1/2 MIC Amp at the indicated incubation periods. (e) Percent survival of clinical isolated MRSA8, MRSA18, S2-lac and TMM2 in the indicated concentrations of Amp plus 20 mM fructose. (f, g) Killing effect of the indicated concentrations of Amp plus 20 mM fructose in treating persisters and bacterial biofilms. (h) Percent survival of zebrafish infected with AR-GBS in the presence or absence of Amp, fructose or both. Results are displayed as mean ± standard errors of the means (SEM) (*N* ≥ 3 technical replicates per sample), and statistically significant differences are identified by t-test. *, *p* < 0.05, **, *p* < 0.01. Each experiment was repeated independently at least three times.

To test the role of fructose on reversing Amp resistance, AR-GBS were incubated with fructose or other biomarkers identified through OPLS-DA. Previous studies showed that metabolites always have best efficacy at molar ratio, and high level (≥10 mM) of glucose, maltose, alanine, glutamine and glycine have no detectable symptoms on animals, we initially test their synergies with Amp at 20 mM [19,22]. Whereas Fructose showed the most potent synergistic effect, which increased the killing of Amp by 773 folds, as compared to other metabolites (Figure 5(a)). This killing effect was fructose dose-dependent (Figure 5(b)) and Amp dose-dependent (Figure 5(c)). In addition, fructose in promoting Amp killing effect was increased along with increasing time (Figure 5(d)). To further corroborate that fructose was potent on promoting Amp killing, clinic isolated multidrug-resistant bacteria including two methicillin-resistant *Staphylococcus aureus* (MRSA) strains, one *Escherichia coli* expressing New Delhi metallo-β-lactamase (NDM-1) (S2-lac) strain and one *S. agalactiae* (TMM2), whose MICs ranged from 0.039 to 1250 μg/mL (Supplementary Fig. S3). MRSA and bacterial containing NDM-1 are notorious for their difficult-to-treat characteristic and alerted by WHO [34]. As expected, fructose plus Amp reversed their resistance and promoted Amp killing (Figure 5(e)). Moreover, bacterial persisters and biofilm-forming bacteria are of big concerns during clinic treatment of bacterial infection since they are highly resistant to antibiotic therapy [35]. Antibiotic or fructose alone failed to eradicate both of persisters and biofilm-forming bacteria (Figure 5(f,g)). However, supplementation of fructose potentiated Amp to kill both of the persisters and biofilm-forming bacteria, which decreased survival for 48.45–697.64 folds and 2.64–396.42 folds in a Amp dose-dependent manner, respectively (Figure 5(f,g)). To further confirm the synergistic effect of fructose and Amp *in vivo*, zebrafish, *Danio rerio*, were challenged with AR-GBS. Bacteria infection caused 20.83% percentage of survival, and fructose or Amp alone only slightly increased the survival rate. However, when *D. rerio* were co-treated with fructose and Amp, the survival rate was increased 41.67% to 50% (Figure 5(h)). Thus, these data demonstrate that fructose promotes Amp killing efficacy both *in vitro* and *in vivo*.

### Fructose reactivates glycolysis to promote Amp killing

**Figure 6.**
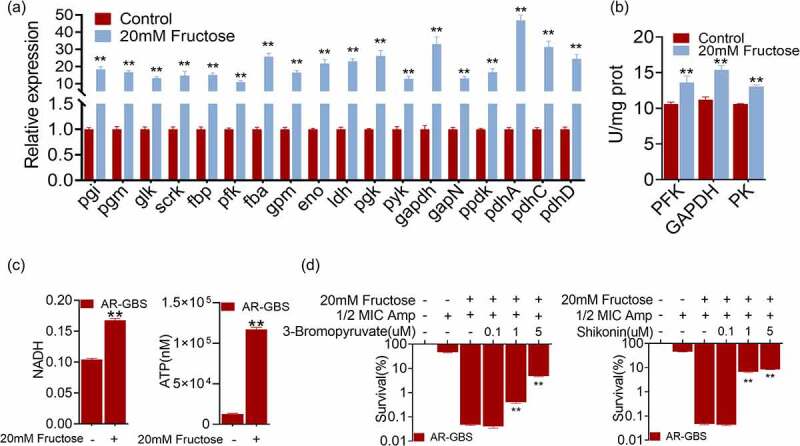
Fructose promotes glycolysis. (a) qRT-PCR for expression of genes in glycolysis in the absence and presence of fructose. (b) Activity of PFK, GAPDH and PK of AR in the absence and presence of fructose. (c) ATP and NADH of AR in the absence and presence of fructose. (d) Percent survival of AR in the presence of fructose plus Amp and effect of 3-bromopyruvate and shikonin. Results are displayed as mean ± standard errors of the means (SEM) (*N* ≥ 3 technical replicates per sample), and statistically significant differences are identified by t-test. *, *p* < 0.05, **, *p* < 0.01. Each experiment was repeated independently at least three times.

To explore the mechanism of fructose in potentiating Amp killing, we quantified the gene expression level of glycolytic pathways. Treatment of AR-GBS with fructose (20 mM) dramatically increased the expression of glycolytic genes (Figure 6(a)). The increased glycolysis was confirmed with enzymatic activity of PFK, GAPDH and PK that were increased 1.28, 1.37 and 1.20 folds, respectively, in the presence of fructose (Figure 6(b)). Moreover, the contents of NADH and ATP were also increased by fructose (Figure 6(c)). These data demonstrate that fructose enhances glycolysis in AR-GBS. To demonstrate that whether glycolysis is responsible for reversing antibiotic resistance, we treated the bacteria with inhibitors 3-bromopyruvic acid and shikonin that targets GAPDH and PK respectively (Supplementary Figure S1). Inhibitors abrogated fructose-potentiated Amp killing for 102–182 folds (Figure 6(d)). These data suggest that activation of glycolysis by fructose is critical to reverse ampicillin resistance in GBS.

### Fructose enhances intracellular accumulation of ampicillin

**Figure 7.**
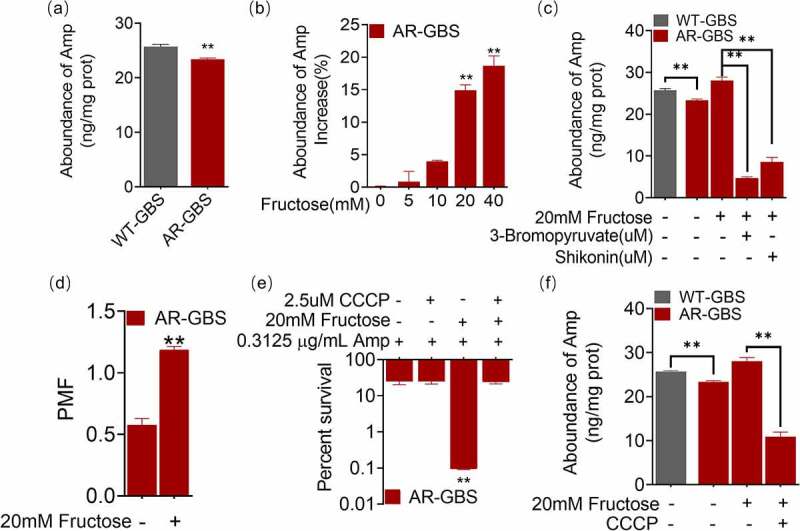
Fructose increases intracellular Amp. (a) Intracellular Amp between WT-GBS and AR-GBS. (b) Intracellular Amp of AR-GBS being treated with indicated concentrations of fructose plus Amp. (c) Intracellular Amp of AR-GBS being treated with 20 mM or fructose plus 3-bromopyruvate or shikonin. (d) PMF of AR-GBS in the absence and presence of fructose. (e) Percent survival of AR-GBS in the presence of fructose plus Amp and effect of m-chlorophenyl hydrazone (CCCP). (f) Intracellular Amp of AR-GBS in the presence of fructose plus Amp or plus CCCP. Results are displayed as mean ± standard errors of the means (SEM) (*N* ≥ 3 technical replicates per sample), and statistically significant differences are identified by t-test. *, *p* < 0.05, **, *p* < 0.01. Each experiment was repeated independently at least three times.

Killing of bacteria by antibiotics is highly dependent on the intracellular concentration of antibiotics. As such, we quantified the intracellular concentration of ampicillin. AR-GBS had lower level of Amp than WT-GBS (Figure 7(a)). Whereas, fructose increased intracellular concentration Amp in a dose-dependent manner, where 20 mM fructose increased 14.89% (Figure 7(b)). However, inhibitors, 3-bromopyruvic acid and shikonin, that abolish glycolytic function, reduced intracellular Amp (Figure 7(c)). Fructose increased PMF that may enhance antibiotic influx. Thus, fructose increased the PMF generations (Figure 7(d)). Inhibition of PMF with CCCP abrogated the killing efficacy of fructose (Figure 7(e)) as well as the intracellular Amp potentiated by fructose (Figure 7(f)). These results suggest that fructose enhances Amp influx dependent on glycolysis and PMF.

### Fructose increased the expression of penicillin binding proteins

**Figure 8.**
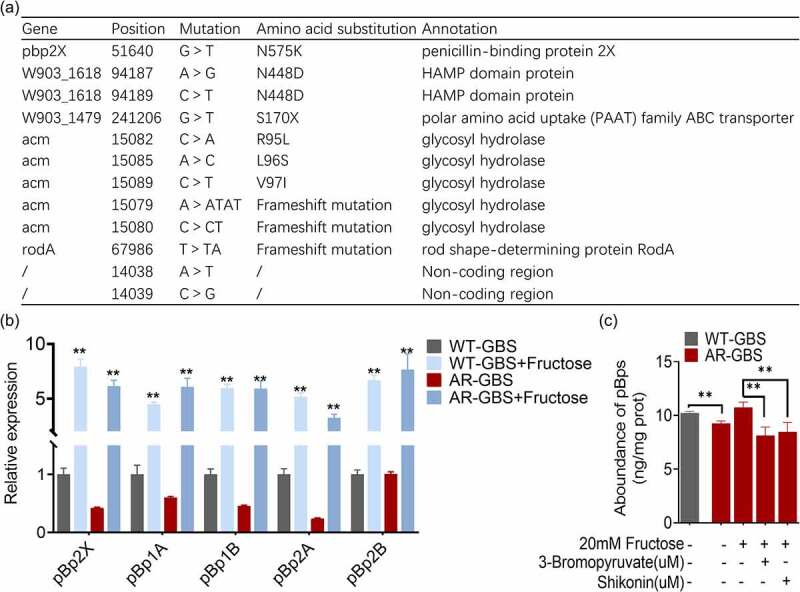
Fructose promotes the expression of pBps. (a) Annotation for mutations of AR-GBS identified from mutants against to WT-GBS. (b) qRT-PCR for expression of pBps in the absence and presence of fructose. (c) the abundance of pBps in WT or AR in the absence and presence of fructose and effect of 3-bromopyruvate and shikonin. Results are displayed as mean ± standard errors of the means (SEM) (*N* ≥ 3 technical replicates per sample), and statistically significant differences are identified by t-test. *, *p* < 0.05, **, *p* < 0.01. Each experiment was repeated independently at least three times.

Ampicillin kills bacteria via directly inhibiting the peptidoglycan synthesis proteins [36]. Mutations of these proteins confer ampicillin resistance in GBS [37]. To better understand the genetic basis underlying ampicillin resistance, we sequenced the whole genome of WT-GBS and AR-GBS. We identified twelve point mutations on AR-GBS as compared to WT-GBS, including two mutations at non-coding region. One frameshift mutation at *rodA* gene, five mutations including point mutation and frameshift mutation on *acm* gene that encodes glycosyl hydrolase, one point mutation on polar amino acid uptake family ABC transporter, two mutations on HAMP domain protein, and one mutation on penicillin-binding protein 2× (pBp2×) (Figure 8(a)). Among the mutated genes, glycoside hydrolase catalyses the hydrolysis of the glycosidic linkage of glycosides, which play roles in shaping bacterial cell fate, signalling and biofilm development in *Pseudomonas aeruginosa* [38]. However, its function on antibiotic resistance and in bacterial physiology of gram-positive bacteria is largely unknown. HAMP domain protein are commonly found as bacterial sensor and chemotaxis proteins, whose mutation is documented to be associated with polymyxin, amikacin and imipenem resistance [39,40]. RodA is a shape, elongation, division and sporulation family glycotransferase, which form complex with pBp2B and exerts transpeptidase and glycosyltransferase activities [41,42]. Mutations on *rodA* increased sensitivity in *Listeria monocytogenes* [43]. But whether it play similar role in GBS needs experimental validation. pBp2× is a known penicillin-binding protein, whose mutation confer penicillin resistance [44–46]. In addition to pBp2×, there were four additional penicillin binding proteins in GBS genome including pBp1A, pBp1B, pBp2A and pBp2B, but find no mutations. Additionally, intrinsic antibiotic resistance in the WT strain was possibly mediated by *bacA* gene, which encodes undecaprenyl pyrophosphate phosphatese, confer bacitracin, a polypeptide antibiotic, resistance. Interestingly, the expression of the five genes except pBp2B was decreased in resistant strains, whereas their expression was exclusively promoted by fructose in both of WT-GBS and AR-GBS (Figure 8(b)). To confirm these results at protein level, pBps including the five proteins were quantified via a commercial available kit that quantify the total penicillin binding proteins instead of individual protein. AR-GBS had lower level of total pBps than WT-GBS, but fructose slightly increased the abundance, which was abolished by inhibitors, 3-bromopyruvic acid and shikonin (Figure 8(c)). Taken together, these studies demonstrate that fructose increased the expression of targets of Amp.

## Discussion

Antibiotic resistance represents a global crisis that requires urgent attention. Despite restricting inappropriate use of antibiotics, actions should also be taken to eliminate the antibiotic-resistant bacteria that threats the sustainable farm husbandry and human health. One of the major approaches to overcome antibiotic resistance is the discovery of novel drugs. However, a very limited number of antibiotics has been approved to be used in clinic in the past decades [47,48]. And unfortunately, no new classes of the antibiotics have been developed in the past fifty years [49]. Although this is an incentive to the pharmaceutic industry to do more research, the development still lags behind. Hence, how to use the currently available antibiotics to resolve infection by antibiotic-resistant bacteria is an urgent topic.

β-lactam antibiotics is one of the primary antibiotics to combat infection by Gram-positive bacteria. The rise of antibiotic resistance to β-lactams in Gram-positive bacteria alert the potential threat [50]. However, the approach in reversing such resistance is not fully investigated. In recent decades, research on bacterial drug resistance from the metabolic level reports that drug resistance is closely related to bacterial metabolism. Repressed central carbon metabolism and energy metabolism represents a characteristic feature of antibiotic-resistant bacteria [51–54]. In this study, we first show that depressed glycolysis is a characteristic feature of antibiotic-resistant GBS, and that can be remodelled with exogenous fructose. This finding is consistent with the concept that bacterial metabolic environment may modulate antibiotic efficacy [55].

Metabolic status of antibiotic-resistant GBS is significantly different from susceptible GBS. One interesting observation here is that the genes expression of glycolysis was decreased, especially *pfk*, *gapdh* and *pyk*. And the specific enzyme activity of enzymes encoded by the three genes were reduced. Being consistent with the enzymatic activity, the content of ATP and NADH was decreased. Streptococcus spp. normally contain incomplete Krebs cycle and oxidative phosphorylation. So energy was largely produced from glycolysis or fermentation by metabolizing carbohydrates from environment [33,56,57]. Glycolysis is one of vital pathways to provide energy for cell activity.

Surprisingly, our study suggests that activation of glycolysis is critical to reverse ampicillin resistance in GBS. And exogenous fructose can reactivate glycolysis. On the one hand, fructose dramatically increased the glycolytic genes as well as enzymatic activity of PFK, GAPDH and PK. Moreover, NADH and ATP were also increased by fructose. On the other hand, fructose increased the killing efficacy of Amp both *in vivo* and *in vitro*, which was fructose dose-dependent. Actually, exogenous fructose activates the TCA cycle, generating NADH and promoting PMF, thereby increases high level of intracellular kanamycin, leading to bacterial death [22]. Unlike the above-mentioned studies, our findings show that fructose fuels glycolysis that generates NADH, serving as donor for generating proton motive force that enhance antibiotic influx. Thus, fructose increased the PMF generations. Furthermore, inhibition of PMF with CCCP can abolish the killing efficacy of fructose (Figure 7(d)) as well as the intracellular amp concentration (Figure 7(e)).

Additionally, unlike the resistant mechanism to β-lactams in Gram-negative bacteria that express hydrolases to destroy the antibiotics, Gram-positive bacteria mainly adopt target modification. As such, in Gram-positive bacteria, penicillin-binding proteins (PBPs) are the targets of β-lactams, which are the primary catalysts for the synthesis and remodelling of the peptidoglycan cell wall of bacteria [36]. Methicillin-resistant *Staphylococcus aureus* express modified PBP or increase PBP production or decrease binding with antibiotics to be resistant to β-lactams [58]. Whereas PBP1a has mutation hotspots in the active site, making β-lactams inaccessible to the binding site in *S. pneumoniae* [59,60]. Our study also demonstrates the mutations on PBPs is involved in ampicillin resistance. PBP mutations are considered the first step in eventual high level of penicillin resistance. However, it is interesting to observe that the increased glycolysis by fructose can exclusively promote the expression of PBPs as well as the intracellular amp concentration.

Interestingly, previous reports suggest the fructose and glucose metabolism are crucial for reversing the antibiotic resistance of cefoperazone/sulbactam-resistant *Pseudomonas aeruginosa*, kanamycin-resistant *Edwardsiella tarda*, and gentamycin-resistant *Vibrio alginolyticus* [22,61,62]. All of the there strains are gram-negative bacteria. And they shared a common mechanism that fructose or glucose need to influx into the pyruvate cycle to promotes the generation of PMF or/and ROS. However, those studies didn’t analyse the involvement of glycolysis, and more importantly, GBS does not possess a complete pyruvate cycle, suggesting glycolysis play critical roles of antibiotic resistance in gram-positive bacteria.

**Figure 9.**
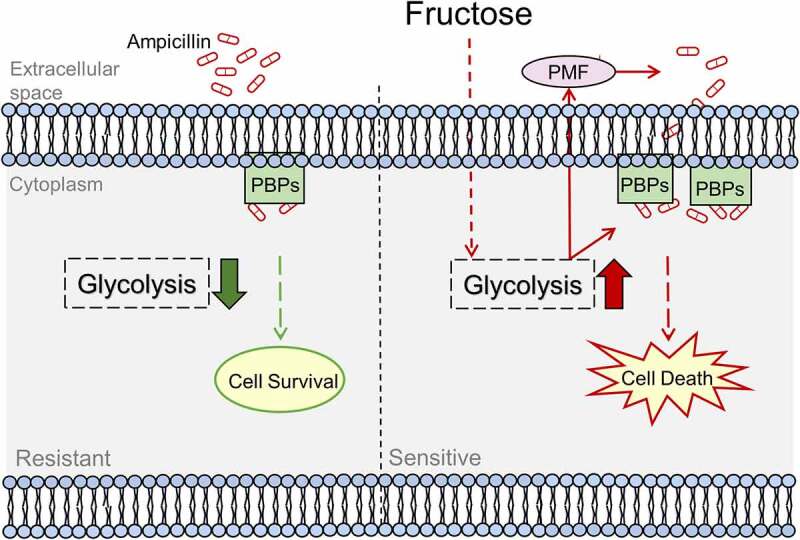
Proposed Model. Ampicillin-resistant GBS has reduced ampicillin uptake that causes low level of intracellular concentration of ampicillin and ampicillin targets, penicillin-binding proteins (PBPs). While exogenous fructose activates glycolysis to enhance ampicillin uptake and PBPs to reverse ampicillin resistance.

In summary, fructose restores glycolysis that increases the influx of ampicillin and expression of penicillin-binding proteins whose expression are repressed in resistant bacteria (Figure 9). In this way, it can be a potent metabolite to be used in combination with ampicillin to eliminate ampicillin-resistant bacteria. This approach may reduce the need to develop new drugs.

## Materials and methods

### Ethics statement

The animal used in this study is zebrafish, *Danio rerio*. All of the experiments involving the use of zebrafish were conducted as instructed by Guide for the Care and Use of Laboratory Animals of the National Institutes of Health. And the protocol adopted in this manuscript has been reviewed and approved by the Institutional Animal Care and Use Committee of Sun Yat-sen University (approval no. SYSU-IACUC-2020-B126716).

### Bacterial strains and culture conditions

The GBS strain used in this study was a general gift from Prof. Li AXE, School of Life Sciences, Sun Yat-sen University. This strain was isolated from a diseased tilapia. To propagate the bacterial growth, a single colony was grown at 37°C in Brian Heart Infusion (BHI) medium. Ampicillin-resistant *S. agalactiae* (AR-GBS) was selected by sequential propagation of *S. agalactiae* in medium in ampicillin. GBS propagated without ampicillin was used as a control.

### Determination of the minimum inhibitory concentration (MIC)

MIC value was determined as previously described [63]. Briefly, overnight GBS culture was inoculated into fresh BHI medium at a ratio of 1:100, and was incubated at 37°C until OD600 was 0.5. Then, 1 × 10^5^ colony forming unit (CFU) of bacteria were aliquoted into 96-well microplate containing serial 2-fold dilutions of antibiotics. The microplate was incubated at 37°C for 16 h. MIC was defined as the lowest antibiotic concentration that inhibited visible growth. Three biological replicates were included for each experiment.

### GC-MS sample preparation and analysis

Preparation of bacterial sample for metabolomic analysis was performed as previously described [64,65]. GBS were harvested by centrifugation, washed and re-suspended in saline buffer until OD600 = 1.0. Then, 10 ml bacteria were aliquoted and immediately quenched with cold methanol, mixed thoroughly and sonicated for 10 min (200 W). The sonicated sample was centrifuged to collect supernatants, which were moved to a new tube containing 10 μL 0.2 mg/mL ribitol, the internal standard to monitor the mass spectrometry performance. The sample was dried by vacuum. Dried samples were treated to 80 μL 20 mg/ml methoxyamine hydrochloride dissolved in pyridine (Sigma – Aldrich) for 180 min at 37◦C. The same volume of N-methyl-N-(trimethylsilyl) trifluoroacetamide (Sigma – Aldrich) was added to the solution and was kept at the same temperature for another 45 min. Samples were cleared by centrifugation and supernatant were collected for gas chromatography-mass spectrometry (GC-MS) analysis. GC-MS was carried out in Agilent G1701EAGC-MSD ChemStation (Agilent). Briefly, 1 μL of the derivatized sample was injected into a dodecyl benzene sulphonic acid (DBS) column (30-m length, 250-μm inner diameter [i.d.], 0.25-μm thickness) column in a split less mode, where injection port had a temperature at 270 ◦C. Electron ionization (EI) is the ion source that provides whose ionization energy was 70 eV and acceleration voltage was 8000 V. For GC-MS analysis, the parameters for GC oven was set as following: 85 ◦C for 3 min, gradual increase to 285 ◦C (5 ◦C/min), then 310 ◦C (20 ◦C/min) and held for 7 min. Helium was the carrier gas, whose flow rate was 1 mL/min. Scan mode was set to 50–600 m/z.

### GC-MS data analysis

Date processing was as previously described [65]. Mass fragmentation spectrum was extracted by XCalibur software (Thermo fisher, version 2.1). To identify compounds, the mass spectrum was used to search against the database of National Institute of Standards and Technology (NIST) library. Data were normalized by the internal standards, and adjusted by the total intensity of all metabolites. Metabolite of significant difference was analysed by IBM SPSS Statistics 19. Heatmap was constructed by R software (version 643.6.1). Differential metabolites were screened by Kruskal-Wallis test and Mann – Whitney test (SPSS 13.0), where *p* < 0.01 was considered significant. Hierarchical clustering was constructed in R by distance matrix. Principal component analysis (PCA) and orthogonal partial least square discriminant analysis (OPLS-DA) were performed in SIMCA 12.0.1 (Umetrics, Umeå, Sweden).

### Pathway enrichment analysis

To enrich pathways for the differential metabolites, an on-line tool, MetaboAnalyst 5.0, was adopted at the website: http://www.metaboanalyst.ca/. Pathways were enriched by p value<0.05 and impact score [65].

### Quantitative real-time polymerase chain reaction (qRT-PCR)

qRT-PCR was performed as previously described [19,66]. Total RNA was isolated from GBS with TRIzol (Invitrogen Life Technologies, United States) after treatment with lysozyme for 30 min at 37℃. The quality of RNA was monitored by 1% electrophoresis and Nanodrop. Reverse transcription was conducted with 1 μg total RNA with EvoM-MLV RT kit with gDNA clean for qPCR (AG11705; Accurate Biology). And qRT-PCR was performed in a reaction volume of 10 μl including 5 μl 2×SYBR green premix pro Taq HS qPCR kit (AG11701; Accurate Biotechnology), 2.6 μl H_2_O, 2 μl cDNA template, and 0.2 μl each of forward and reverse primers (10 mM) in 384-well plates. Primers used in this study was included in Supplementary Table S1. The reactions were set on a LightCycler 480 system (Roche, Germany). The cycling parameters were 95°C for 30 s, 40 cycles of 95°C for 10 s, and 60°C for 30 s. Fluorescence were measured at 72°C for 1 s during each cycle. At last, reaction was terminated at 95°C with a calefactive velocity of 5°C/s to obtain the melting curve. Data are shown as the relative mRNA expression compared with control group and standardized with the endogenous reference 16S rRNA gene. Statistic significance was calculated by 2^−ΔΔCt^ method [67].

### Measurement of the specific activity of glycolytic enzymes

The specific enzyme activity of phosphofructokinase (PFK), glyceraldehyde-3-phosphate dehydrogenase (GAPDH), and pyruvate kinase (PK) was measured according to manufacture’s instruction (Catlog No. KT20327, KT20332 and KT20328, respectively, Moshake, Wuhan, China). WT-GBS or AR-GBS were cultured in BHI, washed three times with sterile saline, and was resuspended in 1× PBS (pH 7.0) to OD600 = 1.0. 5 ml of bacterial suspension was used for enzymatic activity assay that were centrifuged and resuspended in 1 mL phosphate-buffered saline (PBS) containing 10 mg lysozyme. After incubation for 30 min, bacteria were lysed by sonication for 10 min (200 W total power with 35% output, 2s pulse, 3s pause over ice). The solution was then centrifuged with 12,000 rpm at 4°C for 10 min to remove insoluble materials. Protein concentration of the supernatant was quantified by BCA protein concentration determination kit (P0009, Beyotime).

Specific enzymatic activities of PFK/GAPDH/PK activity were quantified by sandwich enzyme-linked immunosorbent assay (ELISA) based on that the antibody recognizes the phosphorylation or acetylation site that is only present in activated enzyme. 10 μL supernatant was added to ELISA plate pre-coated with antibodies against PFK, GAPDH or PK and then combined with HRP-labelled PFK, GAPDH or PK antibody to form antibody-antigen-HRP-conjugated antibody complex. The substrate TMB is converted into blue under the catalysis of HRP enzyme, and the final yellow colour under the action of acid is positively correlated with PFK/GAPDH/PK activity in the sample. The absorbance at 450 nm was measured in a PerkinElmer LS55 Fluorescence Spectrophotometer (PerkinElmer). PFK/GAPDH/PK activity concentration was calculated by standard curve. And the specific activities of the above three enzymes were calculated by normalizing the units of PFK/GAPDH/PK activity with the quantity of protein in each sample, which were expressed as U/mg protein.

### Quantification of ATP

ATP content was determined with BacTiter-GloTM Microbial Cell Viability Assay (Cat. G8231, Promega, Madison, WI, United States) [28]. Briefly, bacterial cells were harvested by centrifugation at 8000 rpm for 3 min, washed and resuspended in saline solution to an OD600 = 0.2. Then, 50 μL of the sample were dispensed into 96-well plate and mixed with an equal volume assay solution. Absorbance were read in PerkinElmer, Turku, Finland. The concentration of ATP was calculated based on the standard curve of ATP.

### Quantification of NADH

NADH was quantified with EnzyChrom NAD/NADH Assay Kit (Catlog. No., BioAssay Systems, U.S.A.). In brief, bacterial cells were resuspended to OD 0.2 and incubated with fructose or/and ampicillin at 37 °C for 6 h. Cells were harvested and resuspended in NADH extraction buffer. After being incubated in 60 °C water for 5 min, cell lysis were added with assay buffer and NAD exaction buffer, and briefly vortexed. Insoluble material was removed by centrifugation, and the supernatant collected for reading 0 min and 15 min value at 565 nm. The concentration of NADH was calculated based on the standard curve of NADH.

### Measurement of membrane potential

To quantify the membrane potential, BacLight Bacterial Membrane Potential Kit (Invitrogen) was used. Bacterial cells at a concentration of 1 × 10^6^ CFU/mL were stained with 10 μL of 2 mM DiOC2 (3). The dye was incubated with bacteria for 30 min at 37 ℃. Then, bacteria were analysed in a FACSCalibur flow cytometer (Becton Dickinson, San Jose, CA). The green/red fluorescence was detected through a 488 − 530/610 nm bandwidth band-pass filter, respectively. Thus, membrane potential was determined by the fluorescence intensity of red to green.

### Preparation of persisters

Preparation of persister was performed according to the established protocol by Keren and Allison et al [68,69]. A single colony was picked from plate and cultured in BHI medium with shaking at 37 °C, 200 rpm. Then the overnight culture (~6 × 10^9^ CFU/mL cells) were treated with 5 μg/mL ciprofloxacin for 4 h to kill growing bacteria, and the resulting bacteria were persisters. To verify persisters, they were treated with increased concentration of ciprofloxacin up to 500 μg/mL, and the viability of bacteria would not drop significantly.

### Preparation of biofilm

Killing of biofilm-forming bacteria was performed as previously described [69]. Briefly, 10 μL overnight culture was inoculated in to a 6-mm PE50 catheter (0.58 mm by 0.96 mm) in 1 mL BHI medium, and the catheter was incubated aerobically at 37°C . Culture medium was changed every 1 days for a total of 3 day. The resulting catheters were washed five times with sterile saline to remove loosely attached cells. Catheters were used for biofilm killing experiments.

### Antibiotic killing assay

GBS were collected by centrifugation at 8000 rpm for 3 min, washed with sterile saline, and suspended in 25% BHI medium in M9 minimal media (containing 10 mM acetate, 1 mM MgSO_4_, and 100 μM CaCl_2)_ until OD600 was 0.2. Fructose or/and antibiotic were added and incubated at 37 °C for 6 h, shaking at 200 rpm. To determine bacterial counts, 100 μL of cultures was removed and then serially diluted. An aliquot of 5 μL of each dilution was plated in BHI agar plates and incubated at 37 °C for 16 h. The colonies were counted, and CFU/mL was calculated.

### Measurement of intracellular ampicillin

Quantification of intracellular ampicillin was performed as previously described [19]. GBS were incubated with ampicillin (0.3125 μg/mL) at 37 °C for 6 h, and then collected by centrifugation. Bacterial pellets were washed three times with saline buffer, and resuspended in sterile to OD600 = 1.0. Then 5 mL of the samples were processed with sonication for 10 min (200 W total power with 35% output, 2s pulse, 3s pause over ice). Supernatants were collected to determine AMP concentration with ELISA (Catlog No. E014A02, Shenzhen Lvshiyuan Biotechnology Co., Ltd.). Briefly, 50 μL supernatant were separately added into 96-well immunoplates (pre-coated with 50 μL antibody), 25 °C for 30 min. Then, microplate was extensively washed five times followed with 100 μL of HRP-labelled anti-rabbit IgG for 30 min at 25 °C. The plates were washed four times, and then 50 μL of substrate A solution and B solution was added to each well and incubated for 15 min at 25 °C in the dark, followed by the addition of stopping solution. The absorbance at 450 nm was measured in a PerkinElmer LS55 Fluorescence Spectrophotometer (PerkinElmer). Triplicate was carried out for each sample.

### Zebrafish infection assay

Zebrafish, 2.5–3 cm average in length and 0.2 ± 0.05 g average in weight, were purchased from the Guangzhou Zebrafish Breeding Corporation. The overnight bacterial culture were diluted 1:100 in fresh BHI medium and grew to OD600 = 1.0. The cells were washed and resuspended in sterile saline buffer (0.85% NaCl). Zebrafish were challenged with 5 μL of 1 × 10^6^CFU GBS via intramuscularly injection. One-hour post-infection, fish were treated with a single dose of AMP (10 mg/kg), fructose (100 mg/kg), or AMP plus fructose (10 + 100 mg/kg) (*n* = 30 for each treatment). Survival of fish were monitored twice a day for a total of 7 days.

### Measurement of penicillin binding proteins (PBPs)

Expression level of penicillin-binding proteins was quantified by ELISA following manufactures’ instruction (Catlog No. KT20326, Wuhan MSKbio). Briefly, bacteria were incubated in M9 minimal media (containing 10 mM acetate, 1 mM MgSO_4_, and 100 μM CaCl_2_) containing 25% BHI medium at 37 °C for 6 h and collected by centrifugation at 8,000 g for 3 min. The resulting pellets were washed three times, resuspended in sterile saline and adjusted to OD1.0. Then 5 mL of the samples were processed with sonication for 10 min (200 W total power with 35% output, 2s pulse, 3s pause over ice), and supernatant were collected. The prepared samples were diluted and separately added into 96-well immunoplates coated with 50 μL antibody, 37 °C for 30 min. The plates were washed five times and then reacted with 100 μL of HRP-labelled anti-rabbit IgG for 30 min at 37 °C. The plates were washed five times, and then 50 μL of substrate A solution and B solution was added to each well and incubated for 15 min at 37 °C in the dark, followed by the addition of stopping solution. The absorbance at 450 nm was measured in a PerkinElmer LS55 Fluorescence Spectrophotometer (PerkinElmer). Triplicate was included for each sample.

### Illumina sequencing and bioinformatics analysis

Overnight bacterial culture, obtained from three colonies of WT-GBS and AR-GBS, were washed 2 times with sterile water. Then, bacteria were lysed by grounding in liquid nitrogen, following treatment with SDS and potassium acetate. DNA was precipitated with isopropanol, following RNase treatment, and DNA was precipitated again. DNA was sheared into~350 bp fragment, ligated with Illumina adaptor, and was sequenced in Illumina NovaSeq 6000 [70]. ANNOVAR was used to identify the mutations from *S. agalactiae* CNCTC 10/84 (W903, Assembly: GCA_000782855.1) and *S. agalactiae 2603* (SAG, Assembly: GCA_000007265.1) [71–73].

### Statistic analysis

Statistical analysis was calculated in GraphPad Prism 9 or SPSS26.0. All data are presented as mean ± SD. Unpaired two-sided *t*-test was used for statistical analysis of two groups, while one-way two-sided ANOVA was used to compare multiple groups. For *in vivo* studies, two-sided Gehan – Wilcoxon test or Mann-Whitney U test unless was used. Differences with *p* < 0.05 were considered significant.

## Authors contribution

BP conceptualized and designed the project. XWC, JHW and YLL conducted the experiments. XWC and JHW performed data analysis. XWC, HMM and BP interpreted the data. BP wrote the manuscript. All the authors reviewed the manuscript and acknowledged the contributions.

## Supplementary Material

Supplemental Material

## Data Availability

The data that support the findings of this study are openly available in this manuscript and supplementary files.
