# Supplementary material for: Fructose promotes ampicillin killing of antibiotic-resistant Streptococcus agalactiae
Source: Virulence. 2023 Feb 26;14(1):2180938. doi: 10.1080/21505594.2023.2180938 (PMC9980678; doi:10.1080/21505594.2023.2180938)
Supplement: Supplemental Material [file KVIR_A_2180938_SM0650.docx]

**Supplementary material**


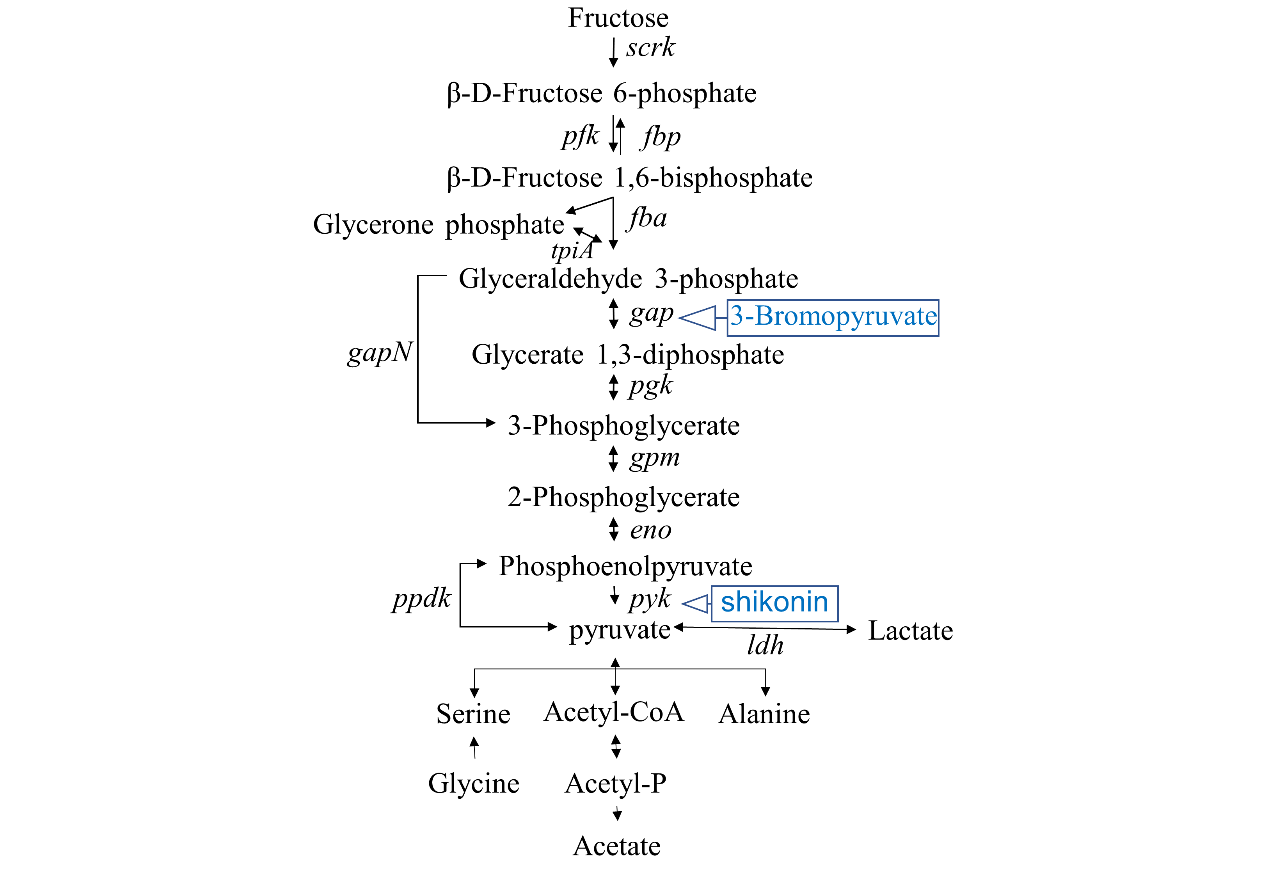


**Figure S1**. Central carbon metabolism in *S. agalactiae,* and the targets of inhibitors


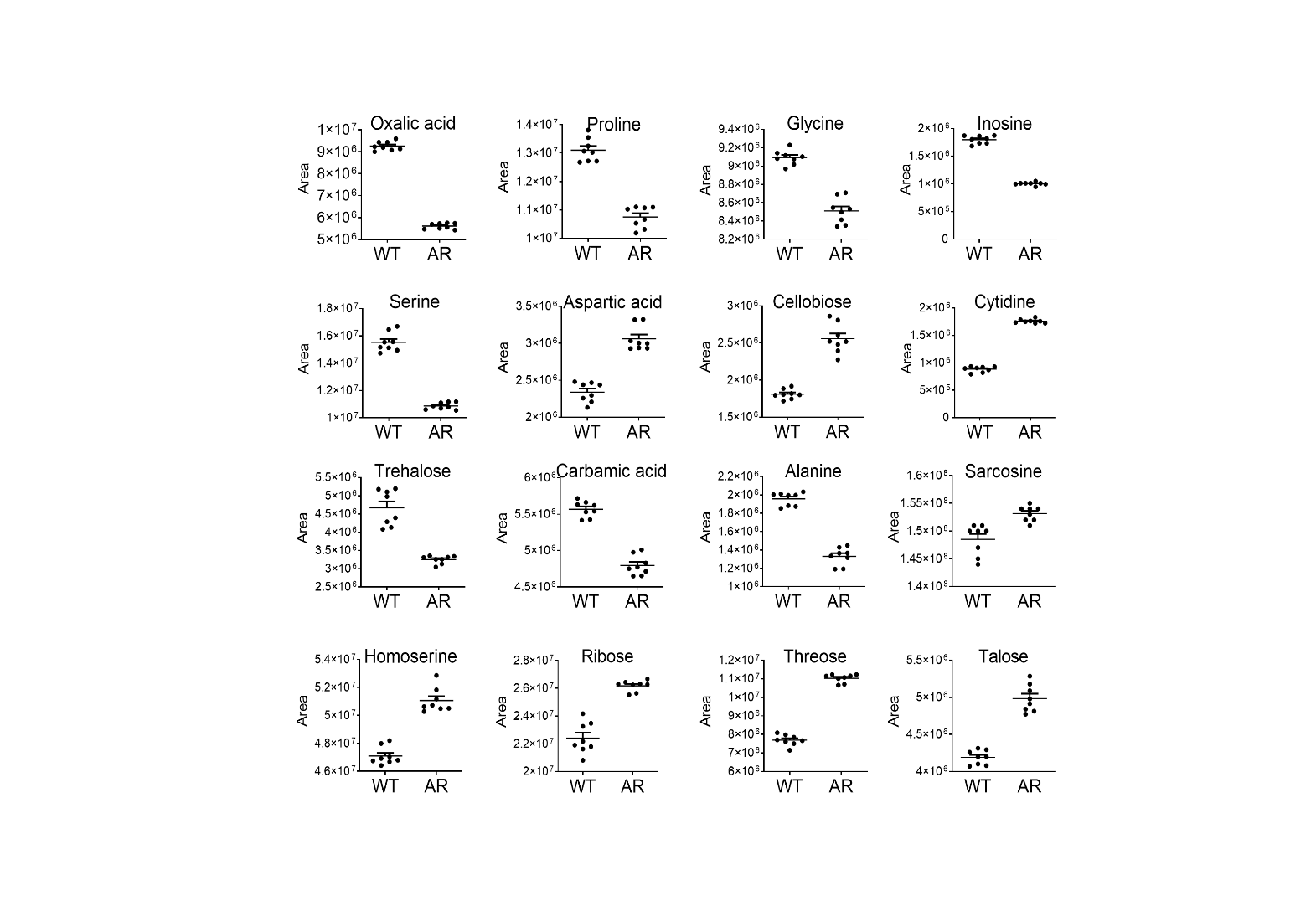


**Figure S2** Comparison of the crucial biomarkers from data (Fig.4B).


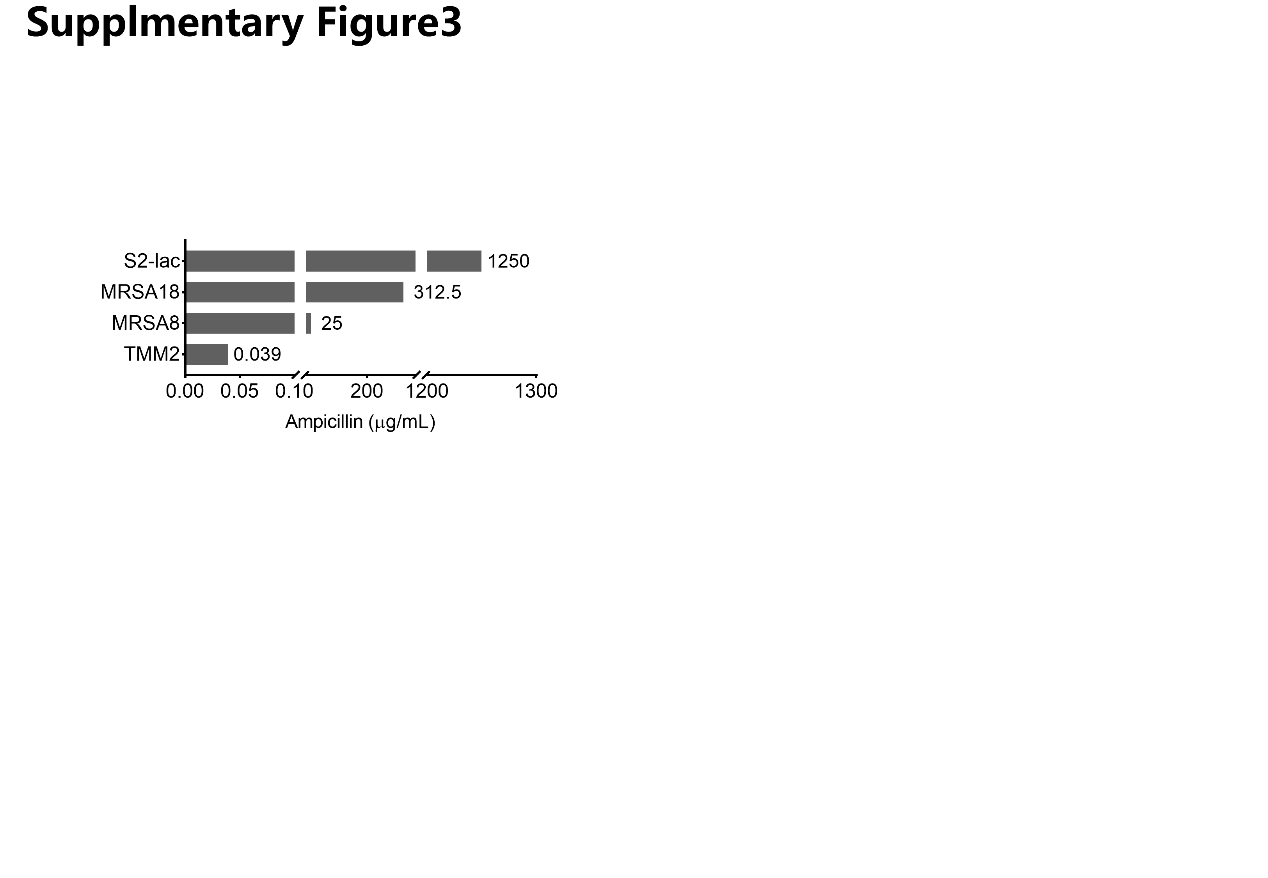


**Figure S3** MIC between MRSA8, MRSA18 and TMM2.

| **Table S1.** Primers used in this study | | |
| --- | --- | --- |
| **Gene** | **Primer** | **Nucleotide Sequence (5′-3′)** |
| *pgi* | Forward | TTGCAAACCGCAGAAGAACG |
|  | Reverse | CGGAAAGCAATCGCTGGTTC |
| *pgm* | Forward | CAAGCAACCGAAGGATTGGC |
|  | Reverse | TCTGGTGTTGGACGAAGAGC |
| *glk* | Forward | AAAGCAGCGATTGACAACGG |
|  | Reverse | CTGCTGCCAGTCCAAGGTAA |
| *scrk* | Forward | ACGACGCCTCAAGAAACGAT |
|  | Reverse | CCAATGCAACTTTGGGGTCG |
| *fbp* | Forward | ACTTGGATGGGAGCAGCATC |
|  | Reverse | ACGGCGTGAGTAATCAACCA |
| *pfk* | Forward | AGTTGCGACAGCAGTTGAGA |
|  | Reverse | CTGCAGCGATACCTGACCAA |
| *fba* | Forward | AACGGCTATGCAGTAGGTGG |
|  | Reverse | TAGCCGCACCCATTGAAGTT |
| *gpm* | Forward | AGCACATACTGATCGTCGCT |
|  | Reverse | AGTTACCGTGTGCACCAACA |
| *eno* | Forward | TCCTTGACTCACGCGGTAAC |
|  | Reverse | TAACGAGACTTGTCGCCGTC |
| *ldh* | Forward | TATAGGCATGGGGCACGTTG |
|  | Reverse | TGACAACGCGTCATAGTCGT |
| *pgk* | Forward | AAGTTCGCGACACTGAAGGT |
|  | Reverse | GACACCCATAGGTCCGTTCC |
| *pyk* | Forward | CGTTGCAACACTTGGTCCTG |
|  | Reverse | CCATACGAGCTCCTTGCTCA |
| *gapdh* | Forward | GTCTTGCATTCCGTCGCATC |
|  | Reverse | ACAGTACCGTCGAAACGACC |
| *gapN* | Forward | AGGACGCGGTTCTGAGATTG |
|  | Reverse | TCAGCTAAGACGACACCTGC |
| *ppdk* | Forward | ACGCTTGCCATATCCGAGAG |
|  | Reverse | CCTGACCGAACTGACACCAA |
| *pdhA* | Forward | TGCGATTGCAGGTTCAGCTA |
|  | Reverse | GTGCAGCCGAACCAATACCT |
| *pdhC* | Forward | CCACTCCTGCAGCTCGTAAA |
|  | Reverse | TACGTGGTTGAGCACCCTTG |
| *pdhD* | Forward | AACGCACTCGGCGGTAATAA |
|  | Reverse | AGCACGACCATTGCCTGTAA |
| *pBp1A* | Forward | ACAATAGCGCCGTTGGTACA |
|  | Reverse | TCCGCCAGTTAGGTAGGACA |
| *pBp1B* | Forward | ACAACGTACACCAGTGGCAA |
|  | Reverse | CGGCTTTGGGAACTACACCT |
| *pBp2A* | Forward | ACAAAGAGCTAGGAGTGGCG |
|  | Reverse | GCGCGATGCATTATACCGTC |
| *pBp2B* | Forward | CGCGCTAGTTTTTCCGAAGG |
|  | Reverse | TCCTGATGATAGCGTCGCAC |
| *pBp2X* | Forward | TTTTTCTACCAGTCCGGGGC |
|  | Reverse | GTTAAGTGCCGCTTCAAGGC |
